# Distinct mechanisms of hypoglycaemia in patients with somatostatin‐secreting neuroendocrine tumours

**DOI:** 10.1002/edm2.83

**Published:** 2019-06-27

**Authors:** Peter Wiesli, Vojtech Pavlicek, Michael Brändle, Thomas Pfammatter, Aurel Perren, Christoph Schmid

**Affiliations:** ^1^ Medizinische Klinik Kantonsspital Frauenfeld Frauenfeld Switzerland; ^2^ Medizinische Klinik Kantonsspital Münsterlingen Münsterlingen Switzerland; ^3^ Department of Internal Medicine Kantonsspital St.Gallen St.Gallen Switzerland; ^4^ Institute of Diagnostic and Interventional Radiology University Hospital of Zurich Zurich Switzerland; ^5^ Department of Pathology University of Bern Bern Switzerland; ^6^ Division of Endocrinology and Diabetes, Department of Internal Medicine University Hospital of Zurich Zurich Switzerland

**Keywords:** hyperinsulinaemia, hypoglycaemia, neuroendocrine tumour, somatostatinoma

## Abstract

**Introduction:**

Somatostatin‐secreting neuroendocrine tumours may present with diabetes, cholelithiasis and steatorrhoea. In addition, hypoglycaemia has been associated with somatostatinomas. However, the mechanism of hypoglycaemia in patients with somatostatinomas has not been well characterized.

**Methods:**

We describe two patients with recurrent neuroglycopenic episodes caused by somatostatin‐secreting neuroendocrine tumours in the liver, detected by abdominal CTs and whole‐body octreotide scintigraphy scans and confirmed by biopsy.

**Results:**

Pancreatic islet hyperplasia and co‐secretion of insulin (in addition to somatostatin) from tumour cells, respectively, have been characterized as completely distinct mechanisms of hypoglycaemia at both the functional and morphological levels in these two patients.

**Conclusions:**

Hypoglycaemia may be caused by different mechanisms in patients with somatostatinomas.

Somatostatinomas are rare neuroendocrine tumours usually located in the pancreas or in the periampullary region of the duodenum, rarely in the jejunum, the ovaries or elsewhere. Patients with somatostatinomas most often present with nonspecific symptoms such as abdominal pain or weight loss. A more specific clinical manifestation is the somatostatinoma syndrome characterized by diabetes, cholelithiasis and steatorrhoea.^1^ Moreover, hypoglycaemia has been associated with somatostatin‐secreting neuroendocrine tumours.^2^, ^3^, ^4^ The pathogenesis of hypoglycaemia in patients with somatostatinoma has not been well characterized. We describe two patients with malignant somatostatinomas who presented with hypoglycaemia. In both patients, the pattern of hypoglycaemia was well characterized and documented at a functional and morphological level.

Patient 1, a 76‐year‐old man, presented at his local hospital with hypoglycaemic episodes occurring exclusively in the late postprandial period, often preceded by a short episode of diarrhoea following carbohydrate‐rich meals. On abdominal CT, he was found to have multiple liver tumour masses, and he was then referred to our clinic for further investigations. Fasting plasma glucose was 7.7 mmol/L. No hypoglycaemic symptoms occurred during a 72‐hour fast (plasma glucose concentration 4.8 mmol/L at the end of the fast). In contrast, venous plasma glucose concentration dropped to 1.5 mmol/L and the patient developed severe neuroglycopenic symptoms 240 minutes after intake of 75 g glucose during an oral glucose tolerance test (oGTT). Insulin concentrations during the oGTT showed an excessive increase from 44 pmol/L at baseline up to 6998 pmol/L within 120 minutes following the oral glucose challenge. An intravenous GTT, however, did not provoke hypoglycaemia. A tumour‐produced incretin could not be identified (we checked for GLP‐1, CCK, pro CCK and GIP), but somatostatin levels were markedly increased in the basal state (5 nmol/L, normal <0.1) and decreased during the glucose challenge. Selective arterial calcium stimulation with hepatic venous sampling (ASVS) was performed, and insulin concentrations were measured as previously described.^5^ ASVS demonstrated increased insulin secretion in response to calcium injection into all arteries supplying the pancreas but not in response to calcium injection into the proper hepatic artery supplying the tumour (Figure 1). Biopsy of the liver tumour was performed, and histological examination revealed a neuroendocrine tumour. A diagnosis of a somatostatin‐secreting neuroendocrine tumour with postprandial hypoglycaemia, mild diabetes mellitus and cholecystolithiasis was made.

**Figure 1:**
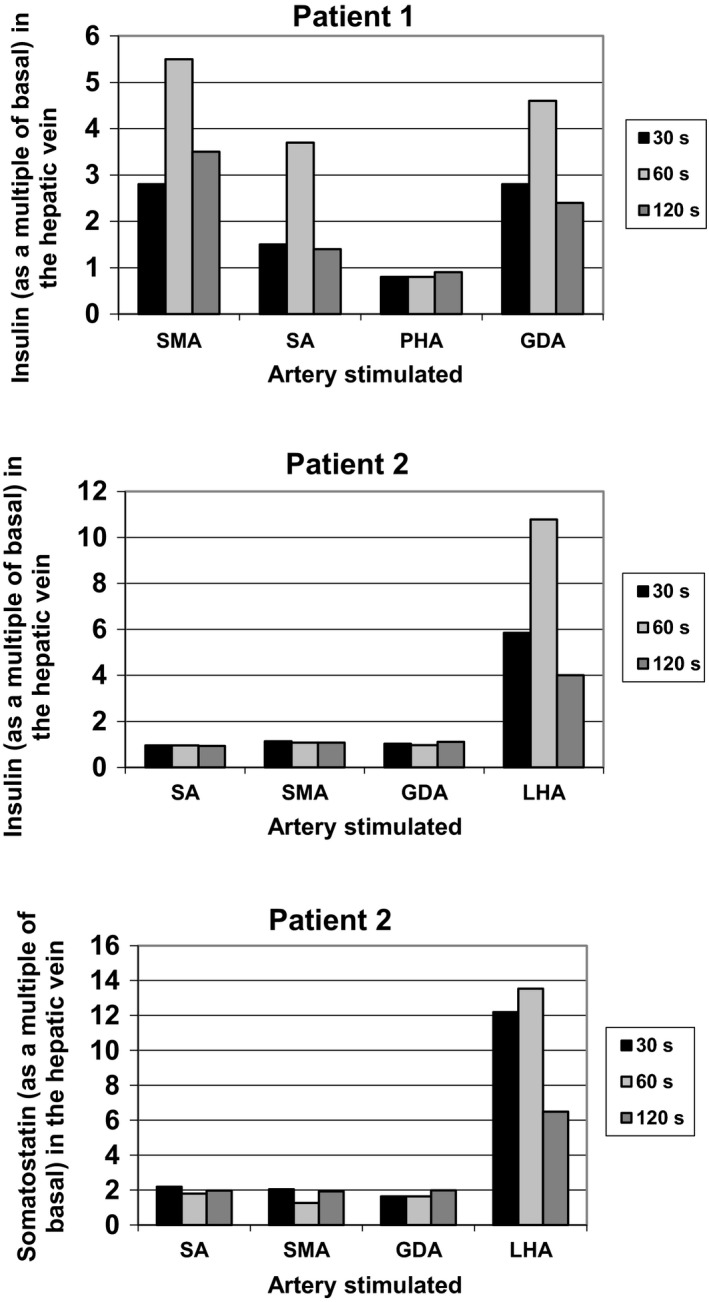
Selective arterial calcium stimulation (ASVS) tests. ASVS was performed as previously described (5). Insulin (and somatostatin for patient 2) levels in the left hepatic vein are shown as a multiple of basal 30, 60 and 120 s after the intraarterial injection of calcium (0.025 mEq Ca^++^ per kg body weight) into arteries supplying the pancreas and the liver (shown in the sequence of injections): in the first patient, the superior mesenteric artery (SMA), the splenic artery (SA), the proper hepatic artery and the (inferior) gastroduodenal artery (GDA); in the second patient, the SA, the SMA, right hepatic artery, the GDA and the left hepatic artery (LHA). A more than twofold rise in the insulin level in the hepatic vein indicates pathological β‐cells in the arterial distribution of the artery stimulated. In patient 1, the pathological increase in insulin levels in the hepatic vein after calcium stimulation of all pancreatic arteries is suggestive for islet cell hyperplasia. In patient 2, the pathological increase in insulin levels following calcium injection into the LHA indicates an insulin‐secreting tumour in the left hepatic lobe

Because the patient declined resection of the right hepatic lobe, transarterial tumour embolization was performed. Postprandial hypoglycaemia improved following the procedure. However, within three years, the neuroendocrine tumour progressed and the patient died at the age of 79 years. Post‐mortem analysis revealed a malignant somatostatinoma with disseminated tumour infiltrates forming nodules up to 10 mm in the pancreas and prominent liver metastasis. The primary localization of the somatostatinoma in the pancreas was not detected until autopsy. In addition to the somatostatinoma, histological examination of the pancreas revealed islet hyperplasia throughout the pancreas. Most strikingly, the number of islets of Langerhans was increased, and their size and shape were very variable with individual hypertrophic islets. Islet hyperplasia as cause of hypoglycaemia in this patient was well characterized at the functional (ASVS test) and morphological level.

Patient 2, a 56‐year‐old woman, presented with recurrent hypoglycaemic episodes. Neuroglycopenic symptoms developed in an erratic manner but preferentially while fasting. In a local hospital, an HbA1c of 4.0%, random low blood glucose readings and a liver tumour by an abdominal CT scan were found. She was sent to us for further investigations and treatment. During a supervised fast, plasma glucose concentration dropped to 1.1 mmol/L after 21 hours. Insulin concentration at the time of hypoglycaemia (458 pmol/L) confirmed hyperinsulinaemic hypoglycaemia. The abdominal CT scan showed a large liver tumour (10 cm in diameter) in the left hepatic lobe. An octreotide scan of the liver tumour was positive (Figure 2). A biopsy of the liver tumour was performed, and histological examination revealed a neuroendocrine tumour positive for synaptophysin and chromogranin A. The differential diagnosis included islet carcinoma of the pancreas metastatic to liver that could not be detected. However, the ASVS test demonstrated a normal insulin secretion response to calcium injection into all arteries supplying the pancreas but a markedly increased (more than 10‐fold) insulin secretion following calcium injection into the left hepatic artery. Moreover, calcium stimulated the secretion of somatostatin by the liver tumour (more than 13‐fold) but not from the pancreas (Figure 1). Resection of the liver tumour was performed. Immunohistochemically, tumour cells stained markedly positive for somatostatin (95%) and insulin (5%); interestingly, staining for the two hormones was restricted to distinct cell populations and not uniform throughout the tumour (Figure 3). Following resection of the liver tumour, insulin fell rapidly whereas plasma glucose levels increased, initially overshooting to an increased level and soon returning into a normal range. The patient remained free of hypoglycaemia and diabetes for the rest of her life. However, manifestations of tumour disease recurred after 3 years, and repeated imaging then revealed progressive hepatic but no pancreatic tumour masses. The patient died 6 years after surgery (at the age of 62 years). Her family members declined a post‐mortem analysis. Evidence for somatostatin secretion by liver tumours is very rare, and usually, somatostatin‐positive neuroendocrine tumours are derived from the pancreas and the duodenum. Co‐secretion of insulin (in addition to somatostatin) by the hepatic somatostatinoma was well characterized as mechanism of hypoglycaemia in this patient.

**Figure 2:**
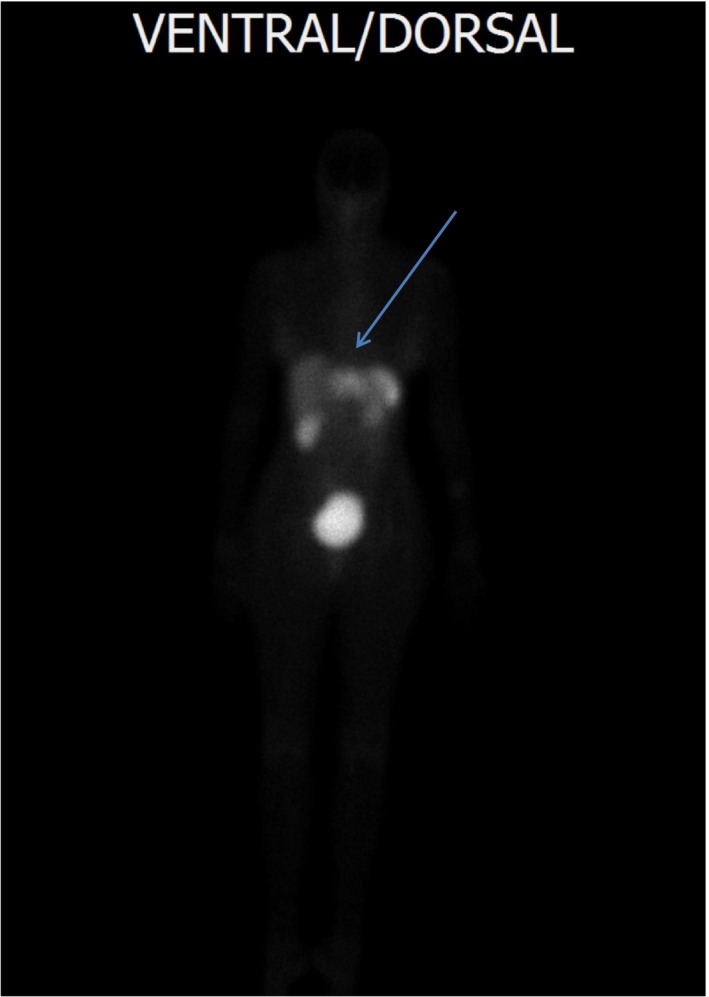
Indium‐111 octreotide scintigraphy. Indium‐111 octreotide scintigraphy of patient 2 revealing increased activity in left hepatic lobe (arrow)

**Figure 3:**
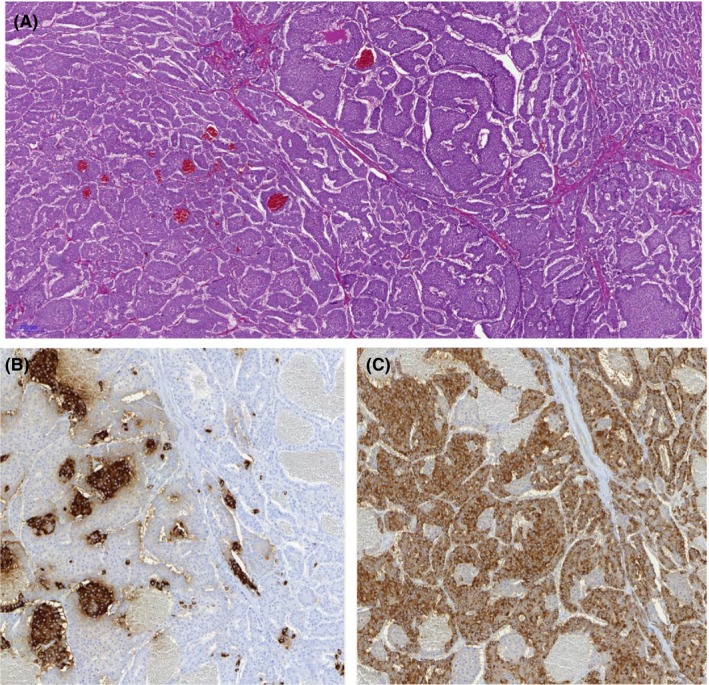
Pathology findings. H&E staining 5× (A) of the liver metastasis, showing a trabecular and nesting growth pattern. Immunohistochemistry for insulin shows a distinct cell population (B 10 × magnification), 10×, compared to immunohistochemistry of somatostatin (C, 10 × magnification). The two cell populations are intermingled, but without different aspect in cell morphology

In conclusion, pancreatic islet cell hyperplasia and co‐secretion of insulin (in addition to somatostatin) from tumour cells, respectively, have been characterized as completely distinct mechanisms of hypoglycaemia at the functional and morphological level in these two patients with malignant somatostatinomas.

## CONFLICT OF INTEREST

The authors declare no conflict of interest in relation to this work.

## AUTHORS’ CONTRIBUTIONS

PW, VP, MB and CS were directly involved in the management of the patients and contributed to the final manuscript. TP performed the ASVS tests, and AP performed the pathological examinations.

## ETHICS STATEMENT

Both patients gave informed consent to publish their data.

## Data Availability

Data sharing is not applicable to this article as no new data are shown. Data to support the findings are available from the corresponding author (PW), upon reasonable request.

## References

[1] Batcher E , Madaj P , Gianoukakis AG . Pancreatic neuroendocrine tumors. Endocr Res. 2011;36(1):35‐43. 21226566 10.3109/07435800.2010.525085

[2] Wright J , Abolfathi A , Penman E , Marks V . Pancreatic somatostatinoma presenting with hypoglycemia. Clin Endocrinol (Oxf). 1980;12(6):603‐608. 6105026 10.1111/j.1365-2265.1980.tb01382.x

[3] Todd JF , Stanley SA , Roufosse CA , et al. A tumour that secretes glucagon‐like‐petptide‐1 and somatostatin in a patient with reactive hypoglycaemia and diabetes. Lancet. 2003;361(9353):228‐230. 12547550 10.1016/s0140-6736(03)12256-8

[4] Sugiyama T , Nakanishi M , Hoshimoto K , et al. Severely fluctuating blood glucose levels associated with a somatostatin‐producing ovarian neuroendocrine tumor. J Clin Endocrinol Metab. 2012;97:3845‐3850. 22962430 10.1210/jc.2012-2091

[5] Wiesli P , Uthoff H , Perren A , et al. Are biochemical markers of neuroendocrine tumors co‐released with insulin following local calcium stimulation in patients with insulinoma? Pancreas. 2011;40(7):995‐999. 21705951 10.1097/MPA.0b013e31821ffce1

